# SSH: A Tool for Predicting Hydrophobic Interaction of Monoclonal Antibodies Using Sequences

**DOI:** 10.1155/2020/3508107

**Published:** 2020-06-02

**Authors:** Anthony Mackitz Dzisoo, Juanjuan Kang, Pengcheng Yao, Benjamin Klugah-Brown, Birga Anteneh Mengesha, Jian Huang

**Affiliations:** ^1^Center for Informational Biology, University of Electronic Science and Technology of China, Chengdu 611731, China; ^2^Brain Connectivity Lab, University of Electronic Science and Technology of China, 611731, China

## Abstract

Therapeutic antibodies are one of the most important parts of the pharmaceutical industry. They are widely used in treating various diseases such as autoimmune diseases, cancer, inflammation, and infectious diseases. Their development process however is often brought to a standstill or takes a longer time and is then more expensive due to their hydrophobicity problems. Hydrophobic interactions can cause problems on half-life, drug administration, and immunogenicity at all stages of antibody drug development. Some of the most widely accepted and used technologies for determining the hydrophobic interactions of antibodies include standup monolayer adsorption chromatography (SMAC), salt-gradient affinity-capture self-interaction nanoparticle spectroscopy (SGAC-SINS), and hydrophobic interaction chromatography (HIC). However, to measure SMAC, SGAC-SINS, and HIC for hundreds of antibody drug candidates is time-consuming and costly. To save time and money, a predictor called SSH is developed. Based on the antibody's sequence only, it can predict the hydrophobic interactions of monoclonal antibodies (mAbs). Using the leave-one-out crossvalidation, SSH achieved 91.226% accuracy, 96.396% sensitivity or recall, 84.196% specificity, 87.754% precision, 0.828 Mathew correlation coefficient (MCC), 0.919 *f*-score, and 0.961 area under the receiver operating characteristic (ROC) curve (AUC).

## 1. Introduction

One of the developing areas in the pharmaceutical industry is therapeutic antibody. The antibody drugs have been used in the treatment of autoimmune diseases, cancer, inflammation, and infectious diseases. However, developing antibody candidates as therapeutic drugs is an expensive and perilous process. Many monoclonal antibody (mAb) candidates failed due to various problems such as poor manufacturability, low stability and solubility, high viscosity, hydrophobicity, and aggregation propensity [1, 2].

Though problems mentioned above are due to various reasons, hydrophobic interactions between antibodies themselves or materials of containers have been shown to be the most predominant one. Currently, the available wet lab methods for measuring the hydrophobic interaction of monoclonal antibodies include standup monolayer adsorption chromatography (SMAC), hydrophobic interaction chromatography (HIC), and affinity-capture self-interaction nanoparticle spectroscopy (AC-SINS). SMAC is used to assess colloidal stability of antibodies under different buffer conditions. Antibodies with colloidal instability may be more likely to have nonspecific interactions, and hydrophobic interactions have been suggested to be the main mode of problematic interactions [3]. HIC is used to evaluate the solubility, viscosity, and serum clearance of antibodies, which are mainly influenced by the hydrophobicity of mAbs [4]. AC-SINS is widely used to detect antibody self-association [5, 6]. Although many physicochemical factors are involved in protein self-association, the presence of hydrophobic moieties on the protein surface is often the primary driver [7]. The methods above have offered a high-throughput solution to developability screening at early-stage antibody drug discovery. However, experimentally screening a large number of candidates is still expensive and time-consuming. Computational screening tools are urgently needed.

Computational methods, especially data mining and machine learning techniques, have been widely used in various aspects of biomedical studies [8–17]. The field of antibody drug development is no exception. There are attempts to predict viscosity, developability, crossinteraction, or self-interaction of antibodies [18–20].

Currently, there are also quite a few existing methods for predicting the hydrophobicity of proteins including mAbs [21–23]. These methods are mostly based on three-dimensional structures of proteins. A recent paper by Jain et al. describes a model for predicting delayed retention of antibodies in HIC from the sequence using machine learning [24]. However, no web service is available for this model, just as other published methods for antibody hydrophobicity prediction.

According to our previous working experience on predicting crossinteraction or self-interaction of antibodies, combining data from different but relevant experimental assays gives better results than just relying on a single experimental assay data. In this study, we combine data from SMAC, SGAC-SINS, and HIC that are closely related to the hydrophobicity of antibodies, build a model using machine learning, and construct a web server called SSH. It can predict hydrophobic interactions of antibodies based on just their sequences. The server is freely available at http://i.uestc.edu.cn/eli/cgi-bin/ssh.pl. We believe it can benefit antibody drug screening community by saving time, money, and resources.

## 2. Results

**Figure 1:**
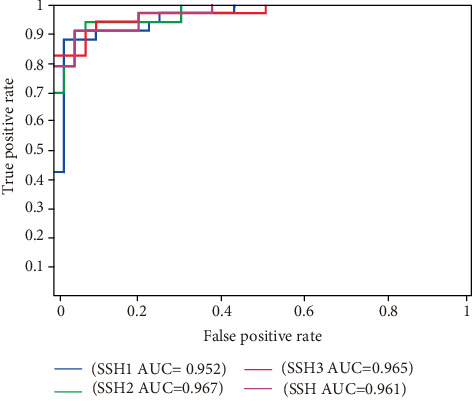
ROC and AUC of our model from the leave-one-out crossvalidation.

The area under the receiver operating characteristic (ROC) curve (AUC), which is a graphical representation of varying threshold values, explains how well a binary classifier can predict the new data. AUC measures the sensitivity and specificity of the binary classification algorithm, which measures the overall performance of the model; it is referred to as how well a model can predict its negative and positive data. Most binary classification uses AUC as a determinant to show how skewed the classification is toward specificity and sensitivity [25]. The analysis of the ROC curve helps to illustrate how well an individual dataset performs independent of the threshold of prediction [26, 27]. As shown in Figure 1 and Table 1, our models achieved AUC of 0.952, 0.967, 0.965, and 0.961 for SSH1, SSH2, SSH3, and SSH, respectively. AUC represented in the ROC curve further indicates good performance of the classifiers.

Also, the sensitivity or true positive rates (TPR) and specificity or false negative rates (FNR) give the discrepancies in the model; it also shows which data and how many positive and negative data are predicted correctly in the leave-one-out crossvalidation. Our ensemble model SSH predicted correctly 96.396% and 84.073% of the positive and negative data, respectively, as shown in Table 1 below.

**Figure 2:**
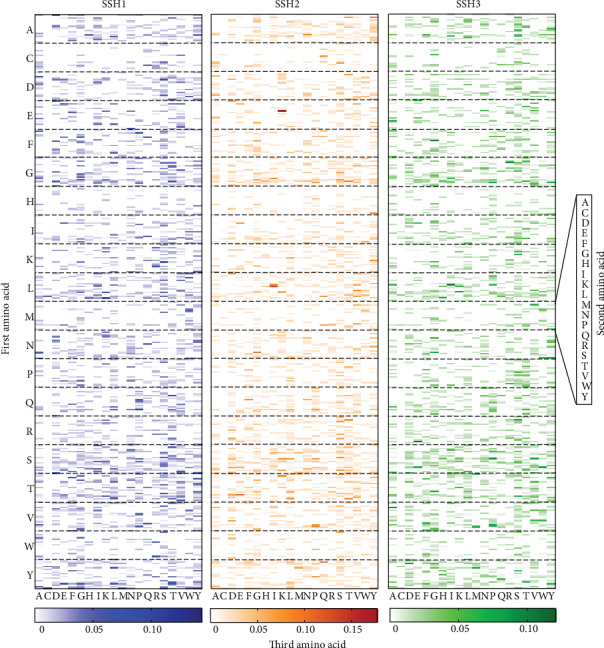
Heat map of the 131 observations in the leave-one-out crossvalidation.

As shown in Figure 2, the heat map from *f*-scores of 8000 tripeptides of the 3 models, SSH1, SSH2, and SSH3, shows which tripeptide or amino acid contributes more to predictive results.

**Figure 3:**
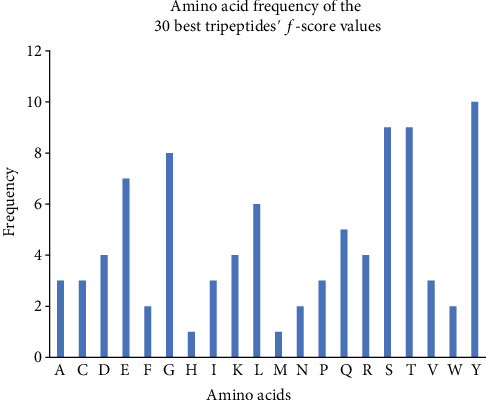
Amino acid frequency from the 30 best tripeptides' *f*-scores.

**Figure 4:**
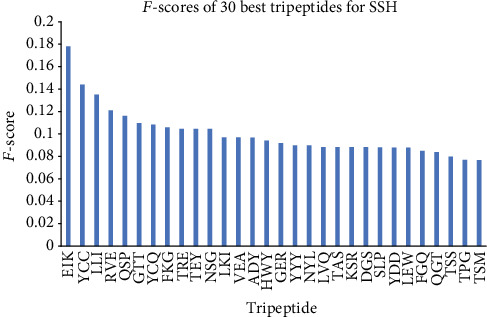
30 tripeptides with the best *f*-scores.

To determine which amino acid gave more predictive values and is the most important to model construction, we calculated the *f*-scores of the tripeptides; the amino acid frequency of the 30 TPC with the best *f*-scores is shown in Figure 3, which shows tyrosine is the most occurring and important in the model construction. Also, Figure 4 shows 30 tripeptides with the best *f*-scores.

## 3. Discussion

In this study, machine learning methods were employed to predict the hydrophobic interactions of antibodies. Improper hydrophobic interactions can cause a lot of problems in antibody drug development. The datasets were constructed according to three biophysical assay values. Our model SSH was trained with TPC and achieved an accuracy of 91.226% using the leave-one-out crossvalidation, with 96.396% sensitivity or recall, 84.100% specificity, 87.754% precision, 0.828 MCC, 0.919 *f*-score, and 0.961 AUC. This work provides the ability to accurately predict flags in antibodies caused by hydrophobic interactions and will help facilitate the ease of development and subsequent drug manufacturing.

From our analysis, tyrosine, serine, threonine, and glycine are the four amino acids with the best *f*-scores or the best predictive amino acids; tyrosine residues are vastly present in the active sites of antibodies [28, 29].

The 96.396% sensitivity proved the ability of our model to correctly identify those antibodies with “flags,” and the 84.10% specificity proved the ability of our model to correctly identify those antibodies without “flags.” The AUC of 0.961 and MCC of 0.828 proved that our model is good at predicting both the negative and positive data.

To determine the prediction results or SSH, a voting method is used depending on the *p* value of the three models SSH1, SSH2, and SSH3. SSH predicts the probability of each antibody input. The higher the probability is, the more likely the antibody is to have hydrophobicity problems. Also, users can set the threshold between 0 and 1, with a higher threshold meaning stricter validation.

In summary, the predictor enhanced our knowledge of how problems in antibodies could be detected for cost and time reduction; also, the work shows the possibility of virtual screening antibody drug candidates in a large scale at the early stage of development.

## 4. Dataset and Methods

### 4.1. Dataset

**Figure 5:**
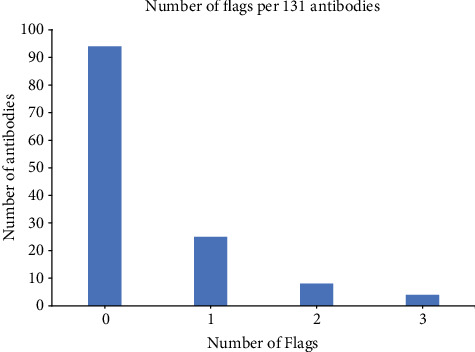
Number of antibodies per flag of 131 antibodies.

The antibody dataset was downloaded from the supplementary materials of the article published by Jain et al. [30]. The dataset includes 48 approved antibodies and 89 antibodies in the phase 2 and phase 3 clinical trials with 6 entries excluded due to conflicting sequences. The remaining 131 antibodies were used to develop SSH. The 10% threshold was employed as in Jain et al. to determine if the antibody has 1 or more “flags” (problems) according to the 3 assays, i.e., SMAC, SGAC-SINS, and HIC [30]. An antibody is labeled with a flag if one of its above assay values falls within the worst 10% threshold. On the other hand, the antibody with an assay value that falls outside the threshold value is deemed without a flag. Of the 131 antibodies, 94 have no flag, 25 have exactly one flag, 8 antibodies have exactly two flags, and 4 antibodies have exactly three flags, as shown in Figure 5. The antibodies with no flags were used as the negative dataset, and those antibodies with at least one flag were used as the positive dataset. The datasets are not balanced, since there are more negative entries. To solve this problem, we split the negative dataset randomly into three subsets with 31, 31, and 32 antibodies, respectively. Each subset is paired with the positive dataset, and 3 models were trained and called SSH1, SSH2, and SSH3. An ensemble method is used to combine the 3 models into SSH using the voting method.

### 4.2. Features and Feature Selection

The tripeptide composition (TPC) is widely used to convert the sequences to vectors as TPC helps to reflect the sequence order and total amino acid composition. TPC has better predictive results than a single amino acid and a dipeptide composition [19, 31]. The method for extracting TPC is shown as

(1) $$\begin{array}{c} \text{TPC}\left(i\right)=\frac{x\left(i\right)}{\sum_{i=1}^{8000}x\left(i\right)}, \end{array}$$

where *i* equals one of the 8000 tripeptide compositions and *x*(*i*) denotes the number of residues of each type of sequence.

From TPC, the best features were selected from the 8000 features using (fselet.py) in LIBSVM, which made use of *f*-scores to obtain the optimal features; given two sets of real numbers, *f*-score technique measures the discrimination of the two sets [32]. Finally, 313, 315, and 315 features were used to build models SSH1, SSH2, and SSH3, respectively.

### 4.3. The Threshold Method

The threshold method is used to generate indexes for grouping the negative and positive datasets, as shown in Table 2. The 10% threshold is calculated as

(2) $$\begin{array}{c} \text{Thresholdval}=\frac{\sum_{i=N}^{i=0}X_{\left(N-y+i\right)}}{y}, \end{array}$$

where *N* is the number of antibodies, *X*(*i*) is the *i*th antibody's assay value, and *y* = 10%(*N*).

### 4.4. Support Vector Machine (SVM)

The support vector machine (SVM) orders data by finding the best hyperplane separating two classes of data points. The best hyperplane for an SVM means the one with the largest margin between the two classes. The margin means the maximal width of the slab parallel to the hyperplane that has no interior data points. SVM is a machine learning method for classifying binary data and multiple class data. It is an effective machine learning method for supervised pattern recognition based on the theory of statistical learning. SVM has been widely used in the field of bioinformatics. We employed LIBSVM [33] with the following parameters: *C* = 2, 128, and 512 and *g* = 0.0078125, 0.0001220703125, and 0.0001220703125 for SSH1, SSH2, and SSH3, respectively, for the development of SSH using “RBF” kernel with the leave-one-out crossvalidation [33] .

### 4.5. Performance Evaluation of SSH

To measure the performance of the SSH, the leave-one-out crossvalidation was used with these measurement parameters, namely, sensitivity (SN), specificity (SP), Mathew correlation coefficient (MCC), accuracy (ACC), and AUC.

Precision is the proportion of the predicted positive cases that were correct. However, accuracy is not only the true measure of a model; the Mathew correlation coefficient (MCC) should be included to evaluate the prediction performance of the developed tool (Equation (6)). MCC is another measure used in machine learning for judging the quality of binary classifications and is considered to be the most robust parameter of any class prediction method.

(3) $$\begin{array}{c} \text{SN}/\text{Recall}=\frac{\text{TP}}{\text{TP}+\text{FN}}, \end{array}$$

(4) $$\begin{array}{c} \text{SP}=\frac{\text{TN}}{\text{TN}+\text{FP}}, \end{array}$$

(5) $$\begin{array}{c} \text{ACC}=\frac{\text{TP}+\text{TN}}{\text{TP}+\text{FN}+\text{TN}+\text{FP}}, \end{array}$$

(6) $$\begin{array}{c} \text{MCC}=\frac{\text{TP}*\text{TN}-\text{FP}*\text{FN}}{\sqrt{\left(\text{TP}+\text{FP}\right)\left(\text{TP}+\text{FN}\right)\left(\text{TN}+\text{FP}\right)\left(\text{TN}+\text{FN}\right)}}, \end{array}$$

where TP is true positive, FN is false negative, TN is true negative, and FP is false positive.

**Figure 6:**
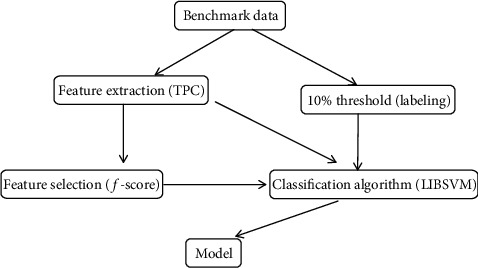
Benchmark of SSH.

Figure 6 shows the benchmark of the SSH; the 10% threshold method is used for labeling the negative and positive data.

## Figures and Tables

**Table 1:** Statistical results of the SSH.

	SSH1	SSH2	SSH3	SSH
Recall/sensitivity	97.297%	94.595%	97.297%	96.396%
Specificity	83.871%	87.097%	81.300%	84.073%
Accuracy	91.177%	92.647%	89.855%	91.226%
BAC	0.906	0.908	0.893	0.902
AUC	0.952	0.967	0.965	0.961
MCC	0.827	0.855	0.803	0.828

**Table 2:** Threshold values of 3 assays [30].

Assays	Threshold values	Units (flags)
Standup monolayer adsorption chromatography (SMAC)	12.8	Retention time (min) (>)
Salt-gradient affinity-capture self-interaction nanoparticle spectroscopy (SGAC-SINS)	370	Salt concentration (mM) (<)
Hydrophobic interaction chromatography (HIC)	11.7	Retention time (min) (>)

## Data Availability

The data used to support the findings of this study are available freely at http://i.uestc.edu.cn/eli/sshdownload.html.

## Abbreviations

AUC: Area under the receiver operating characteristic curve
CDR: Complementarity-determining regions
HIC: Hydrophobic interaction chromatography
MCC: Mathew correlation coefficient
ROC: Receiver operating characteristic curve
SGAC-SINS: Salt-gradient affinity-capture self-interaction nanoparticle spectroscopy
SMAC: Standup monolayer adsorption chromatography
SVM: Support vector machine
TPC: The tripeptide composition
BAC: Balance accuracy.
